# Effect of modified no‐touch laparoscopic radical hysterectomy on outcomes of early stage cervical cancer: A retrospective cohort study

**DOI:** 10.1002/cam4.4612

**Published:** 2022-02-13

**Authors:** Fangjie He, Songhua Yuan, Xia Chen, Siyou Zhang, Yubin Han, Tiecheng Lin, Bingnan Xu, Shimin Huang, Zhiyin Pan

**Affiliations:** ^1^ Department of Obstetrics and Gynecology The First People's Hospital of Foshan Foshan China; ^2^ State Key Laboratory of Oncology in South China Sun Yat‐sen University Cancer Center Guangzhou China

**Keywords:** cervical cancer, enclosed colpotomy, laparoscopic surgery, radical hysterectomy, uterine manipulator

## Abstract

**Objectives:**

We aimed to compare the prognosis of modified no‐touch laparoscopic radical hysterectomy (MLRH) and laparoscopic radical hysterectomy (LRH) on survival in patients with early stage cervical cancer.

**Materials and Methods:**

The clinicopathological data of patients with stage IB1 and IIA1 cervical cancer, who underwent radical surgery between 2014 and 2019, were retrospectively reviewed. The 5‐year disease‐free survival (DFS) and overall survival (OS) were compared between the MLRH and LRH groups using the Kaplan–Meier method. Independent prognostic factors for 5‐year DFS and OS were identified using multivariate, forward, stepwise Cox proportional hazards regression models.

**Results:**

A total of 223 patients with stage IB1 and IIA1 cervical cancer were included. Kaplan–Meier analysis revealed that the 5‐year DFS and OS rates in the MLRH (*n* = 81) group were significantly higher than those in the LRH group (*n* = 142) (DFS, 94.5% vs. 78.8%, *p* = 0.007; OS, 96.7% vs. 87.6%, *p* = 0.033). No significant differences were identified between the two groups in terms of operative time, blood loss, transfusion requirement, and intraoperative or postoperative complications. MLRH was an independent prognostic factor associated with increased 5‐year DFS (adjusted hazard ratio [HR], 0.202; 95% confidence interval [CI], 0.069–0.594; *p* = 0.004) and 5‐year OS (adjusted HR, 0.163; 95% CI, 0.035–0.748; *p* = 0.020).

**Conclusion:**

The oncologic outcomes were superior with MLRH than with LRH in patients with stage IB1 and IIA1 cervical cancer. Contact of cervical tumor cells with the pelvic cavity likely explains the worse prognosis associated with LRH.

## Graphical abstract

The oncologic outcomes were superior with modified no‐touch laparoscopic radical hysterectomy than with laparoscopic radical hysterectomy in patients with stage IB1 and IIA1 cervical cancer. Contact of cervical tumor cells with the pelvic cavity likely explains the worse prognosis.

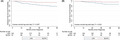

## 1 INTRODUCTION

Radical surgery is the primary treatment for early stage cervical cancer. Laparoscopic radical hysterectomy (LRH) and pelvic lymphadenectomy were initially performed by Nezhat in 1992 and have rapidly evolved since that time.^1^ However, in 2018, two high‐quality studies reported by the *New England Journal of Medicine* revealed that minimally invasive surgery was associated with increased tumor recurrence and decreased survival rates.^2^, ^3^ Therefore, the National Comprehensive Cancer Network® guidelines for cervical cancer suggest open surgery as the gold standard treatment for patients with early stage cervical cancer.^4^

Several potential reasons may explain the increased tumor recurrence caused by laparoscopic surgery, including intracorporeal colpotomy, the use of a uterine manipulator, and the creation of a CO_2_ pneumoperitoneum.^5^, ^6^, ^7^, ^8^ In 1998, Dr. Guangyi Li, first performed LRH to treat patients with early stage cervical cancer in mainland China.^9^ This technique gradually replaced open surgery and became a conventional operation at our institution due to its minimally invasive construct.^10^ In 2013, Dr. Songhua Yuan, the corresponding author of the current study, realized that intracorporeal colpotomy may increase the risk of tumor spillage into the peritoneal cavity; since then, a modified no‐touch laparoscopic radical hysterectomy (MLRH) has been performed routinely for early stage cervical cancer. This technique is similar to vaginal‐assisted LRH,^11^ but is easier to perform, especially for surgeons who lack experience with transvaginal surgery, and has a lower risk of bladder injury. Nonetheless, whether MLRH can decrease tumor spillage and effect of outcomes is unclear and needs to be studied.

This study aimed to determine the effect of MLRH on the outcomes of patients with early stage cervical cancer and reveal the relationship between intracorporeal colpotomy and tumor recurrence.

## 2 MATERIALS AND METHODS

### 2.1 Patients

This single‐center retrospective study was conducted in accordance with the principles of the Declaration of Helsinki and approved by the institutional review board of the First People's Hospital of Foshan (L2021‐1), which waived the need for informed consent because of the retrospective nature of this study. The clinical data of patients who underwent radical surgery for early stage cervical cancer between January 1, 2014 and December 31, 2019 at our institution were collected. Patients were included if they met the following criteria: (1) stage IB1 or IIA1 cervical cancer according to the 2009 International Federation of Gynecology and Obstetrics staging system^12^; (2) diagnosis of squamous cell carcinoma, adenocarcinoma, or adenosquamous carcinoma of the cervix; (3) patients who underwent a type B or C laparoscopic hysterectomy and bilateral pelvic lymphadenectomy (Querleu‐Morrow classification); and (4) MLRH performed by Dr. Songhua Yuan and LRH performed by two other surgeons with similar work experience. Patients were excluded if they received neoadjuvant chemotherapy, conization, or were lost to follow‐up.

A total of 223 patients were included in this study. Patients were divided into two groups based on the surgery type: the MLRH group (*n* = 81) and the LRH group (*n* = 142).

### 2.2 Surgical procedures

Both MLRH and LRH have the same surgical approach for lymphadenectomy, partly the same approach for hysterectomy, and different approaches for colpotomy. The surgical procedures are described herein. First, carbon dioxide was injected into the abdomen to create a pneumoperitoneum, and the pressure was maintained at 12 mmHg. Second, a laparoscope was inserted through the umbilical port. Another 10 mm trocar was inserted at the left midclavicular line approximately 1–2 cm below the umbilical level. Two accessory 5 mm trocars were inserted bilaterally, 3 cm medial to the anterior superior iliac spine. Third, the bilateral pelvic lymph nodes, including the common iliac, external iliac, internal iliac, obturator, and deep inguinal lymph nodes, were removed.^9^, ^10^ Lymph nodes were placed in a bag to prevent port‐site metastases. Lastly, radical hysterectomy was performed with the use of a uterine manipulator, according to the following resection sequence: the round ligament, adnexal pedicle, sacral ligament, cardinal ligament, and vesicocervical ligament.

**FIGURE 1:**
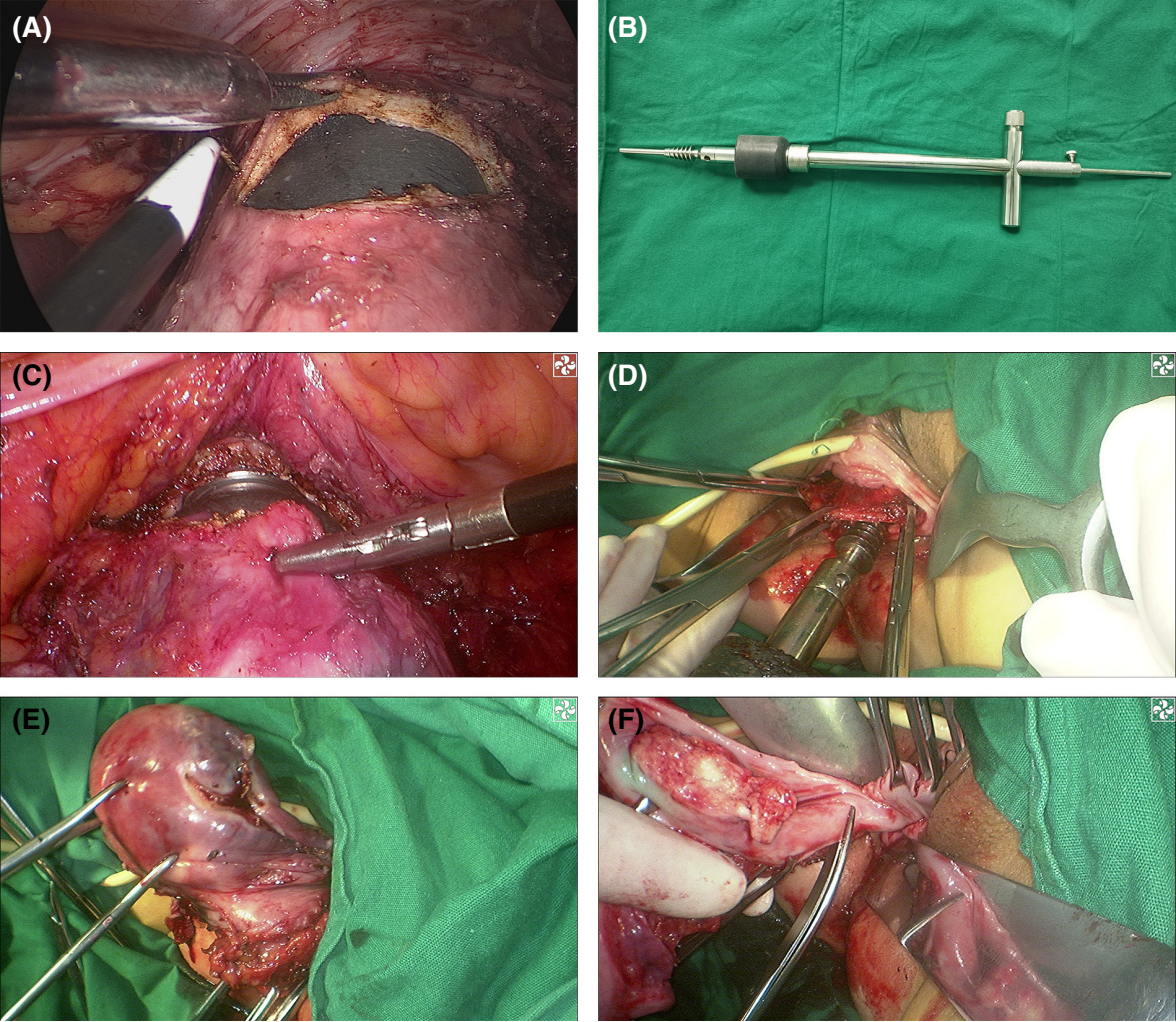
Procedures for removing the uterus during modified no‐touch laparoscopic radical hysterectomy. (A) The anterior wall of the vagina is cut laparoscopically. (B) A uterine manipulator. (C) The anterior wall of the vagina is clamped, and the uterus is pushed together with the uterine manipulator. (D) The anterior wall of the vaginal stump is clamped. (E) The uterine corpus is rolled over and out of the vaginal introitus. (F) The posterior wall of the vagina is cut under direct vision

The main difference between MLRH and LRH is the incision pattern of the colpotomy. LRH conventionally involves circumferentially cutting the vagina completely through a laparoscope. However, tumor cells on the surface of the cervix may touch the pelvic cavity during LRH. In contrast, MLRH involves only cutting the anterior wall of the vagina using a laparoscope (Figure 1A). Then, forceps are used to clamp the anterior wall of the vagina and push the uterus together with the uterine manipulator (Figure 1B) toward the vagina (Figure 1C). Additionally, the CO_2_ pneumoperitoneum is terminated. The following procedures are performed during transvaginal surgery. Three Allis forceps are used to clamp the anterior wall of the vaginal stump, the uterine manipulator is removed (Figure 1D), the anterior wall of the uterine corpus is fixed using two towel forceps, and the uterus is rolled over and out of the vaginal introitus (Figure 1E). The posterior wall of the vagina is cut under direct vision (Figure 1F), and the uterus is subsequently removed. The vaginal stump is closed using continuous hemstitch sutures. Finally, normal saline is employed for vaginal and pelvic cavity lavage to prevent the retention of any residual tumor cells. Normal saline is prevented from flowing into the upper abdomen during pelvic lavage.

### 2.3 Adjuvant therapy

Adjuvant radiotherapy with or without chemotherapy was administered to patients according to the presence of high‐risk factors (positive resection margin, lymph node metastasis, and parametrial involvement) or ≥2 intermediate‐risk factors (stromal invasion depth ≥1/2 and lymphovascular space invasion). This may be influenced by patient and physician preferences.

### 2.4 Follow‐up and endpoint

Patients were followed up until November 30, 2020 through an outpatient information inquiry and examination report system. The primary endpoint was the 5‐year disease‐free survival (DFS), calculated as the number of months from the date of diagnosis to the first evidence of recurrence or death from cervical cancer. The second primary endpoint was overall survival (OS), calculated as the number of months from the date of diagnosis to death from any cause. Additionally, perioperative and postoperative complications were analyzed.

### 2.5 Statistical analysis

The Student's *t*‐test and chi‐squared test were used to compare continuous and categorical variables, respectively, between the two groups. Five‐year DFS and OS were estimated using the Kaplan–Meier method, and differences between the two groups were compared using the log‐rank test. Multivariate, forward, stepwise Cox regression analysis was used to identify independent risk factors for 5‐year DFS and OS. All statistical analyses were performed using STATA ver. 15.0 (StataCorp 2017, Stata Statistical Software: Release 15; StataCorp LLC). Two‐sided *p*‐values <0.05 were considered reflective of statistical significance.

## 3 RESULTS

### 3.1 Patient characteristics

The patient flowchart is shown in Figure S1. The clinical and pathological characteristics of the patients are summarized in Table 1. Of the 223 patients enrolled in the study, 81 underwent MLRH and 142 underwent LRH.

**TABLE 1:** Preoperative clinical characteristics and immunohistochemistry findings of patients with MLRH or without LRH

Characteristic	Total	MLRH	LRH	*p*
Number (%)	223 (100.0)	81 (36.3)	142 (63.7)	
Age, mean (SD), years	49.6 (9.2)	49.6 (9.5)	49.6 (9.0)	0.978
Stage				0.048
IB1	208 (93.3)	72 (88.9)	136 (95.8)	
IIA1	15 (6.7)	9 (11.1)	6 (4.2)	
Tumor diameter, cm				0.993
≤2	77 (34.5)	28 (34.6)	49 (34.5)	
2.1–4	146 (65.5)	53 (65.4)	93 (65.5)	
Histological type, *n* (%)				0.173
Squamous cell carcinoma	181 (81.2)	71 (87.7)	110 (77.5)	
Adenocarcinoma	38 (17.0)	9 (11.1)	29 (20.4)	
Adenosquamous carcinoma	4 (1.8)	1 (1.2)	3 (2.1)	
Grade, *n* (%)				0.239
G1	11 (4.9)	3 (3.7)	8 (5.6)	
G2	158 (70.9)	62 (76.5)	96 (67.6)	
G3	49 (22.0)	13 (16.0)	36 (25.4)	
Unknown	5 (2.2)	3 (3.7)	2 (1.4)	
Stromal invasion depth				0.519
≤1/2	111 (49.8)	38 (46.9)	73 (51.4)	
>1/2	112 (50.2)	43 (53.1)	69 (48.6)	
LVSI				0.822
No	166 (74.4)	61 (75.3)	105 (73.9)	
Yes	57 (25.6)	20 (24.7)	37 (26.1)	
PMI				0.466
No	215 (96.4)	77 (95.1)	138 (97.2)	
Yes	8 (3.6)	4 (4.9)	4 (2.8)	
RMI				0.714
No	215 (96.4)	79 (97.5)	136 (95.8)	
Yes	8 (3.6)	2 (2.5)	6 (4.2)	
LNM				0.684
No	187 (83.9)	69 (85.2)	118 (83.1)	
Yes	36 (16.1)	12 (14.8)	24 (16.9)	
Adjuvant therapy				0.832
No	156 (70.0)	57 (70.4)	99 (69.7)	
Yes	67 (30.0)	24 (29.6)	43 (30.3)	

*Note:* During 223 patients, 30 (13.5%) patients, including 29 patients with LNM and 1 patients with PMI, have revised the staging after the surgery.

Abbreviations: LNM, lymph node metastasis; LRH, laparoscopic radical hysterectomy; LVSI, lymphovascular space invasion; MLRH, modified no‐touch laparoscopic radical hysterectomy; PMI, parametrial involvement; RMI, resection margin involvement; SD, standard deviation.

The clinical and pathological characteristics of patients in the MLRH and LRH groups are also shown in Table 1. No significant differences were identified between the two groups.

### 3.2 Long‐term outcomes

The median follow‐up period for all patients was 40 months, and the median follow‐up for patients who underwent MLRH and LRH was 41 and 38 months, respectively. Among patients who underwent MLRH, four (4.9%) had evidence of recurrence and two (2.5%) died. Among patients who underwent LRH, 25 (17.6%) had evidence of recurrence and 14 (9.9%) died. The patients who underwent MLRH had a lower rate of pelvic recurrence than did those who underwent LRH, even though no significant difference was observed between the two groups (Table S1).

**FIGURE 2:**
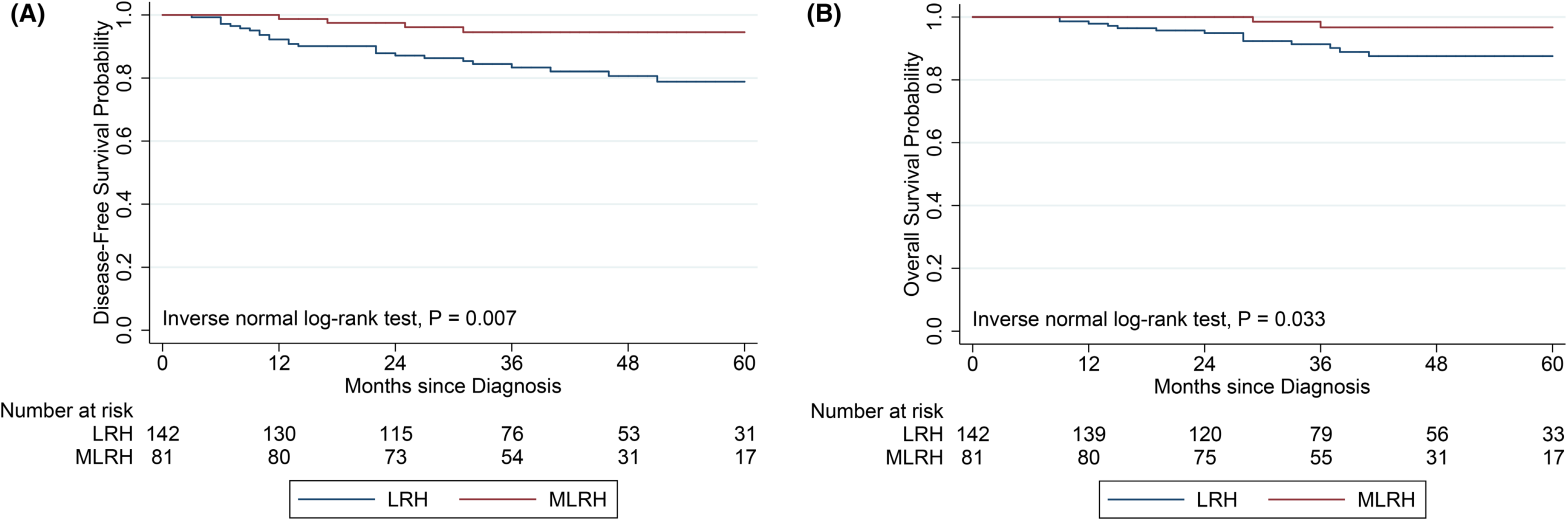
Kaplan–Meier analysis in the modified no‐touch laparoscopic radical hysterectomy and laparoscopic radical hysterectomy groups. (A) Five‐year disease‐free survival. (B) Five‐year overall survival

Compared with patients who underwent LRH, patients who underwent MLRH had a significantly longer 5‐year DFS and OS (5‐year DFS, 94.5% vs. 78.8%, *p* = 0.007; 5‐year OS, 96.7% vs. 87.6%, *p* = 0.033; Figure 2A,B).

On multivariate analysis of the prognostic factors that were identified as significant on univariate analysis (Tables 2 and 3), MLRH was found to be an independent prognostic factor for increased 5‐year DFS (adjusted hazard ratio [HR], 0.202; 95% confidence interval [CI], 0.069–0.594; *p* = 0.004) and 5‐year OS (adjusted HR, 0.163; 95% CI, 0.035–0.748; *p* = 0.020).

**TABLE 2:** Univariate and multivariate analyses of prognostic factors for 5‐year DFS by Cox proportional hazards regression models

Risk factors	Univariate analysis		Multivariate analysis	
	OR (95% CI)	*p*	aOR (95% CI)	*p*
Age	0.984 (0.945–1.025)	0.451	–	0.336
Stage	2.432 (0.845–7.000)	0.099	–	0.084
Tumor diameter	3.747 (1.303–10.772)	0.014	–	0.071
Histologic type	1.495 (0.769–2.907)	0.236	–	0.651
Grade	1.795 (1.008–3.197)	0.047	1.985 (1.073–3.671)	**0.029**
Operation				
LRH	1		1	
MLRH	0.263 (0.092–0.756)	0.013	0.208 (0.069–0.623)	**0.005**
Stromal invasion depth	1.863 (0.876–3.961)	0.106	–	0.587
LVSI	2.557 (1.211–5.402)	0.014	–	0.360
PMI	7.778 (2.944–20.545)	**<**0.001	6.393 (2.052–19.917)	**0.001**
RMI	2.321 (0.550–9.789)	0.251	–	0.881
LNM	4.511 (2.140–9.505)	**<**0.001	3.117 (1.364–7.121)	**0.007**
Adjuvant therapy	4.085 (1.937–8.615)	**<**0.001	–	**–**

*Note:* Adjuvant therapy did not enter in multivariate analysis because of multicollinearity with LNM. The other listed covariates did not have multicollinearity.

Abbreviations: CI, confidence interval; HR, hazard ratio; LNM, lymph node metastasis; LRH, laparoscopic radical hysterectomy; LVSI, lymphovascular space invasion; MLRH, modified no‐touch laparoscopic radical hysterectomy; PMI, parametrial involvement; RMI, resection margin involvement. Bold values indicate independent risk factors of prognosis.

**TABLE 3:** Univariate and multivariate analyses of prognostic factors for 5‐year OS by Cox proportional hazards regression models

Risk factors	Univariate analysis		Multivariate analysis	
	OR (95% CI)	*p*	aOR (95% CI)	*p*
Age	1.006 (0.952–1.063)	0.838	–	0.472
Stage	1.945 (0.442–8.561)	0.379	–	0.591
Tumor diameter	4.258 (0.967–18.751)	0.055	–	0.296
Histologic type	1.497 (0.614–3.651)	0.375	–	0.312
Grade	2.118 (0.994–4.510)	0.052	–	0.141
Operation				
LRH	1		1	
MLRH	0.263 (0.092–0.756)	0.054	0.163 (0.035–0.748)	**0.020**
Stromal invasion depth	1.976 (0.715–5.457)	0.189	–	0.508
LVSI	3.100 (1.142–8.415)	0.026	–	0.875
PMI	13.271 (4.601–38.279)	**<**0.001	7.376 (2.094–25.974)	**0.002**
RMI	4.624 (1.047–20.423)	0.043	–	0.667
LNM	8.430 (3.123–22.760)	**<**0.001	4.358 (1.382–13.740)	**0.012**
Adjuvant therapy	8.911 (2.857–27.790)	**<**0.001	–	**–**

*Note:* Adjuvant therapy did not enter in multivariate analysis because of multicollinearity with LNM. The other listed covariates did not have multicollinearity.

Abbreviations: CI, confidence interval; HR, hazard ratio; LNM, lymph node metastasis; LRH, laparoscopic radical hysterectomy; LVSI, lymphovascular space invasion; MLRH, modified no‐touch laparoscopic radical hysterectomy; PMI, parametrial involvement; RMI, resection margin involvement. Bold values indicate independent risk factors of prognosis.

### 3.3 Short‐term outcomes

The operative details and intraoperative and postoperative complications are shown in Table 4. No significant differences were identified between the two groups in terms of operative time, blood loss, transfusion requirement, and intraoperative and postoperative complications.

**TABLE 4:** Operative details and intraoperative complications

	MLRH (*n* = 81)	LRH (*n* = 142)	*p*
Operative time (min), mean (SD)	258.8 (81.2)	252.9 (76.9)	0.591
Blood loss (ml), mean (range)	292.0 (316.3)	230.5 (219.3)	0.124
Transfusion required, *n* (%)	12 (14.8)	13 (9.2)	0.198
Intraoperative complications, *n* (%)			
Bladder injury	2 (2.5)	4 (2.8)	1.000
Ureter injury	2 (2.5)	2 (1.4)	0.623
Rectal injury	0 (0.0)	0 (0.0)	–
Great vessel injury	0 (0.0)	1 (1.2)	0.185
Obturator nerve injury	0 (0.0)	0 (0.0)	–

Abbreviations: LRH, laparoscopic radical hysterectomy; MLRH, modified no‐touch laparoscopic radical hysterectomy.

## 4 DISCUSSION

To the best of our knowledge, no study has explored the effect of MLRH on the outcomes of patients with early stage cervical cancer and determined the relationship between intracorporeal colpotomy and tumor recurrence. Herein, we found that patients with stage IB1 and IIA1 cervical cancer who underwent MLRH had a better 5‐year DFS and OS than did those who underwent LRH. Furthermore, MLRH and LRH showed no significant differences in operative details or intraoperative complications.

Previous studies have demonstrated that compared with open surgery, laparoscopic surgery is associated with higher pelvic tumor recurrence and worse prognosis in patients with early stage cervical cancer, although the reasons for this are unclear.^2^, ^3^ A few previous studies revealed that intracorporeal colpotomy may be the main reason for decreased survival rates because it can increase the risk of tumor spillage into the peritoneal cavity.^13^, ^14^, ^15^, ^16^, ^17^ However, these studies also abandoned the use of uterine manipulators during surgery. Therefore, whether avoiding the use of uterine manipulators, avoiding intracorporeal colpotomy, or both, results in better prognosis, remains inconclusive. In the current study, we used a uterine manipulator as usual because it can facilitate the operation of radical hysterectomy. However, intracorporeal colpotomy was modified such that cervical tumor cells did not touch the pelvic cavity. The results showed that this modified operation had a lower tumor recurrence rate and a higher survival rate than did conventional LRH. Hence, our study demonstrated that the contact of cervical tumor cells with the pelvic cavity could be one of the main reasons for tumor recurrence, and that LRH was more likely to present with pelvic recurrence, although the difference was not statistically different.

MLRH has several advantages over vaginal‐assisted LRH. First, surgeons can perform a colpotomy of the anterior wall under direct vision through the laparoscope, and the posterior wall of the vagina can be cut under direct vision after the uterus is extracted from the vagina. However, vaginal‐assisted LRH is performed while being blinded to the bladder. Hence, the MLRH approach introduced in this study had a lower risk of intraoperative bladder injury and subsequent requirement for a gynecologist. Second, MLRH reduces operative time because it does not require the formation of a closed vaginal cuff before radical hysterectomy.

The tumor‐free concept of MLRH not only involves the prevention of contact between cervical tumor cells and the pelvic cavity, but also follows certain practices throughout the surgical procedure. First, the manipulator should not penetrate the uterus; it is recommended to fix the uterine manipulator under the direct vision of the laparoscope to avoid perforation. In the MLRH group, three patients in whom the uterus was penetrated were sutured in time to wash the wounds and pelvic cavity with normal saline. There were no recurrences noted in these three patients. Second, the lymph nodes should be collected and placed in bags. Third, normal saline should be used for vaginal and pelvic cavity lavage to prevent retention of residual tumor cells. Finally, MLRH should not be used in patients with tumors larger than 4 cm, or when the uterus cannot be removed through the vagina intact.

The present study could not explain whether the use of a uterine manipulator or CO_2_ pneumoperitoneum was the reason for tumor recurrence in patients with cervical cancer, although the uterine manipulator was suspected to increase the propensity for tumor spillage. However, a recent study reported that the oncologic prognosis associated with uterine manipulator use was superior to that without its use, and multivariate analysis revealed that the uterine manipulator was not an independent risk factor for tumor recurrence.^18^ Furthermore, CO_2_ pneumoperitoneum has been found to promote tumor cell growth or spread in cell‐culture and animal trials.^7^, ^8^ Nonetheless, no clinical trials on patients have been reported. In this study, patients with cervical cancer who underwent MLRH by Dr. Songhua Yuan had a satisfactory prognosis, and the 5‐year DFS and OS were comparable with those of open surgery, as reported in the Laparoscopic Approach to Cervical Cancer Trial (5‐year DFS, 94.5% vs. 96.5%; 5‐year OS, 96.7% vs. 97.6%).^2^ Some studies reported 5‐year DFS and OS of 89.3%–97.7% and 95.2%–99.4%, respectively, in patients with early stage cervical cancer by laparotomy.^19^, ^20^, ^21^ The long‐term prognoses of the patients in our study who underwent MLRH were not inferior to those described in the above studies. Moreover, several studies with high levels of evidence have revealed that laparoscopic surgery is not inferior to open surgery in patients with gastric or colorectal cancer in terms of long‐term survival.^22^, ^23^, ^24^ Therefore, the uterine manipulator and CO_2_ pneumoperitoneum are less likely to be associated with tumor recurrence and patient prognosis.

This study has several limitations. First, this was a retrospective nonrandomized study with the potential for selection bias, even though no significant difference was found between the two groups. Second, this study could not determine whether MLRH was inferior to open radical surgery because of the limited number of cases of open radical surgery performed in the same period at our institution. However, it could reveal that contact of the cervical tumor with pelvic cavity is probably the reason for tumor recurrence. Last, although the surgeons for the two groups had similar numbers of working years and experience, bias due to surgeon experience could not be excluded completely.

## 5 CONCLUSIONS

The present study revealed that MLRH was superior to LRH in terms of a lower risk of tumor recurrence and higher survival rate. Contact of cervical tumor cells with the pelvic cavity may explain the worse prognosis associated with LRH. MLRH is a simple operative technique that needs to be validated in future studies.

## CONFLICT OF INTEREST

None.

## AUTHOR CONTRIBUTIONS

Fangjie He: Writing—original draft, Methodology, Formal analysis. Songhua Yuan: Conceptualization, Writing—original draft, Writing—review & editing, Funding acquisition. Xia Chen: Methodology, Investigation. Siyou Zhang: Methodology, Resources. Yubin Han: Resources, Data curation. Tiecheng Lin: Resources, Data curation. Bingnan Xu: Resources, Data curation. Shimin Huang: Supervision. Zhiyin Pan: Supervision. All authors read and approved the final manuscript.

## ETHICS STATEMENT

This retrospective study was approved by the Institutional Review Board of the First People's Hospital of Foshan (L2021‐1).

## Supporting information


Figure S1

Table S1

## Data Availability

The data that support the findings of this study are available from the corresponding author, Songhua Yuan, upon reasonable request.
