# Supplementary material for: Effect of modified no‐touch laparoscopic radical hysterectomy on outcomes of early stage cervical cancer: A retrospective cohort study
Source: Cancer Med. 2022 Feb 13;11(11):2224–32. doi: 10.1002/cam4.4612 (PMC9160811; doi:10.1002/cam4.4612)
Supplement: Supplementary file 1 — Figure S1 [file CAM4-11-2224-s001.zip › CAM4_4612_supplementary materials.docx]

**Supplementary Materials**

**1.1 Supplementary Figure S1** Flowchart of patient selection.

**1.2 Supplementary Table S1**. Sites of recurrence.

| Site of recurrence | MLRH (n=4) | LRH (n=25) | p |
| --- | --- | --- | --- |
| Site of recurrence |  |  | 0.766 |
| Vault | 1 (25.0) | 5 (20.0) |  |
| Pelvis | 1 (25.0) | 12 (48.0) |  |
| Abdomen | 0 (0.0) | 1 (4.0) |  |
| Distant | 2 (50.0) | 7 (28.0) |  |

MLRH, modified no-touch laparoscopic radical hysterectomy; LRH, laparoscopic radical hysterectomy.
