# Supplementary figures and images for: Effect of modified no‐touch laparoscopic radical hysterectomy on outcomes of early stage cervical cancer: A retrospective cohort study
Source: Cancer Med. 2022 Feb 13;11(11):2224–32. doi: 10.1002/cam4.4612 (PMC9160811; doi:10.1002/cam4.4612)

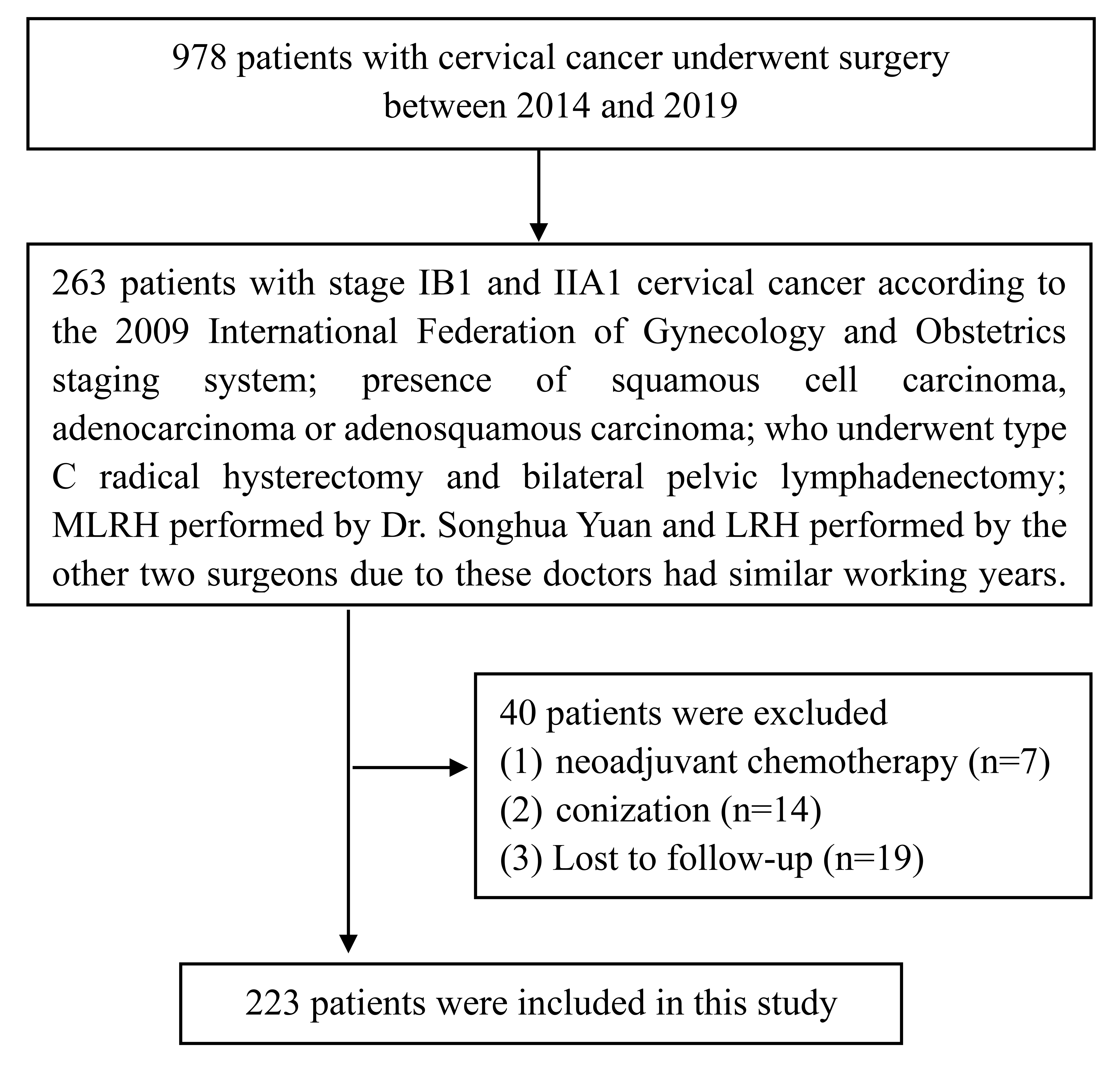

Supplement: Supplementary file 1 — Figure S1 [file CAM4-11-2224-s001.zip › cam44612-sup-0001-FigureS1.tif]
